# Bioactive Natural Products of Marine Sponges from the Genus *Hyrtios*

**DOI:** 10.3390/molecules22050781

**Published:** 2017-05-11

**Authors:** Nourhan Hisham Shady, Ebaa M. El-Hossary, Mostafa A. Fouad, Tobias A. M. Gulder, Mohamed Salah Kamel, Usama Ramadan Abdelmohsen

**Affiliations:** 1 Department of Pharmacognosy, Faculty of Pharmacy, Deraya University, Universities Zone, P.O. Box 61111 New Minia City, Minia, Egypt; noura_shady2013@yahoo.com; 2 National Centre for Radiation Research & Technology, Egyptian Atomic Energy Authority, Ahmed El-Zomor St. 3, El-Zohoor Dist., P.O. Box 29 Nasr City, Cairo, Egypt; ebaa.elhossary@gmail.com; 3 Department of Pharmacognosy, Faculty of Pharmacy, Minia University, 61519 Minia, Egypt; m_fouad2000@yahoo.com (M.A.F.); mskamel@yahoo.com (M.S.K.); 4 Department of Chemistry and Center for Integrated Protein Science Munich (CIPSM), Technische Universität München, Lichtenbergstraβe 4, 85748 Garching, Germany; tobias.gulder@ch.tum.de; 5 Department of Botany II, Julius-von-Sachs Institute for Biological Sciences, University of Würzburg, Julius-von-Sachs-Platz 3, 97082 Würzburg, Germany

**Keywords:** bioactive, marine natural products, marine sponges, *Hyrtios*, alkaloids

## Abstract

Marine sponges are known as a rich source for novel bioactive compounds with valuable pharmacological potential. One of the most predominant sponge genera is *Hyrtios*, reported to have various species such as *Hyrtios erectus*, *Hyrtios reticulatus*, *Hyrtios gumminae*, *Hyrtios communis*, and *Hyrtios tubulatus* and a number of undescribed species. Members of the genus *Hyrtios* are a rich source of natural products with diverse and valuable biological activities, represented by different chemical classes including alkaloids, sesterterpenes and sesquiterpenes. This review covers the literature until June 2016, providing a complete survey of all compounds isolated from the genus *Hyrtios* with their corresponding biological activities whenever applicable.

## 1. Introduction

Marine ecosystems contain enormous, still unexplored and taxonomically diverse macro- and microorganisms. These marine organisms are able to produce novel molecules with large structural diversity and various interesting pharmacological activities [1,2,3,4]. Since 1985, more than 4000 marine natural products possessing a wide range of biological activities have been isolated and characterized [5]. About twenty-four marine natural products are currently in phase I–III clinical trials [6,7]. Moreover, there are currently eight marine natural products on the market, possessing different pharmacological activities [8]. Sponges (phylum Porifera) are among the oldest multicellular animals with a fossil record dating back to Precambrian times [9]. Sponges are widespread in tropical reefs in a great abundance, but can also be found in polar latitudes and the deep, sea as well as in fresh water lakes and rivers [10]. Marine sponges continue to attract attention as rich sources of structurally novel secondary metabolites that are potential lead compounds for the development of new drugs [11]. With more than 280 new isolated compounds from sponges reported during 2014, sponges have returned again to be a superior source of new biologically active marine natural products [11].

Sponges belonging to the genus *Hyrtios* (Kingdom: Animalia, phylum: Porifera, class: Demospongiae, order: Dictyoceratida, family: Thorectidae) are reported to be rich sources of bioactive secondary metabolites. Among marine sponges of the genus *Hyrtios*, the sponge *H. erectus* is the most frequently investigated source of bioactive natural products. For example, several indole alkaloids [12,13], β-carboline alkaloids [14,15] and sesterterpenes [16,17] were isolated from this *Hyrtios* species. Biological investigations of alkaloids and sesterterpenes isolated from *H. erectus* revealed that some of these compounds possess noteworthy anticancer [18,19] and antimicrobial [20,21] activities. The marine sponge *H. erectus* has been collected from different marine environments, including the Red Sea in Egypt [13,16] and Okinawa in Japan [18,22]. The Indonesian marine sponge *H. reticulatus* is another frequently studied marine sponge of this genus reported as a good source of novel β-carboline alkaloids [23,24,25].

In addition, natural product discovery projects from the three sponges *H. gumminae*, *H. communis* and *H. tubulatus* (which has been allocated to the new genus *Dysidea tubulata*) have been reported in the literature. *H. gumminae* was collected from the Andaman Sea in Thailand and was found to be a source of novel sesterterpenoids [26]. Moreover, biological and chemical investigations of the extracts of several undescribed species of the genus *Hyrtios* can be found in the literature. Novel derivatives of puupehenone, a sesquiterpene-methylene quinone, [27,28,29] and alkaloids [30,31,32,33,34,35,36] were the most predominant isolated compounds from the undescribed species of the genus *Hyrtios*. Here, we are reporting an overview on the chemical structures of marine natural products isolated from diverse marine sponges of the genus *Hyrtios*, together with their isolation sources as well as their biological activities whenever applicable.

## 2. *H. erectus*

**Figure 1:**
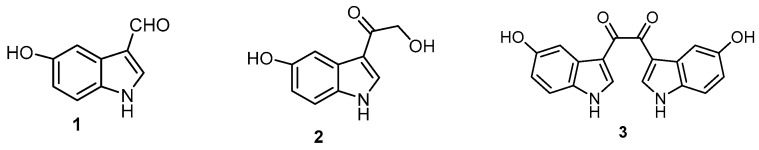
Chemical structures of 5-hydroxyindole-3-aldehyde (**1**), hyrtiosin A (**2**) and hyrtiosin B (**3**).

5-Hydroxyindole-3-aldehyde (**1**) together with the related compounds hyrtiosins A (**2**) and B (**3**) were isolated from the Okinawan marine sponge *H. erectus* collected near Ishigaki Island (Figure 1). Compound **1** showed in vitro cytotoxic activity against human epidermoid carcinoma KB cells with IC_50_ (half maximal inhibitory concentration, concentration causing 50% of the desired activity) value of 4.3 µg/mL, while hyrtiosins A (**2**) and B (**3**) were less cytotoxic than **1** [18].

**Figure 2:**
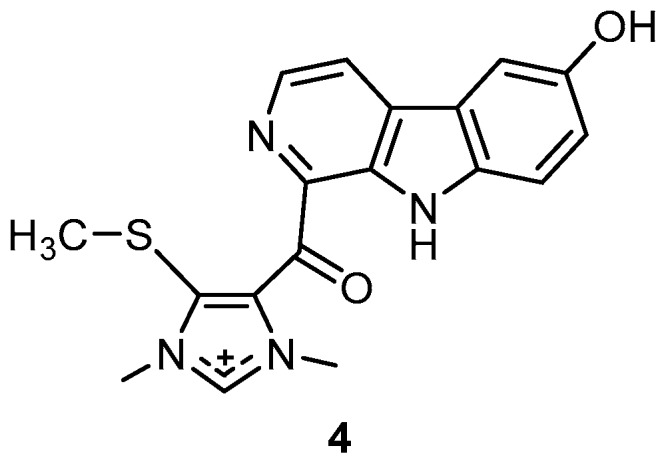
Chemical structure of hyrtiomanzamine (**4**).

Hyrtiomanzamine **4**, a β-carboline alkaloid, was isolated from the marine sponge *H. erectus* collected in the Red Sea (Figure 2). Compound **4** exhibited immunosuppressive activity with an EC_50_ (half maximal effective concentration, concentration causing 50% of the desired activity) of 2 µg/mL in the B lymphocytes reaction assay and no cytotoxic activity on KB cells was observed [37].

**Figure 3:**
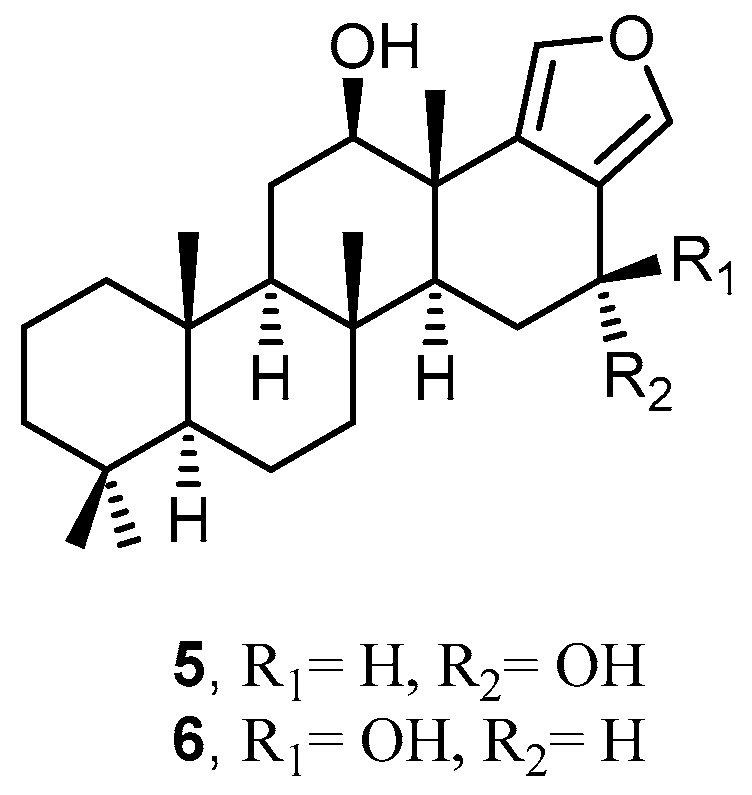
Chemical structures of sesterstatin 4 (**5**) and sesterstatin 5 (**6**).

Two pentacyclic sesterstatins, sesterstatins 4 (**5**) and 5 (**6**), were isolated from the marine sponge *H. erectus* collected in the Republic of Maldives (Figure 3). Compounds **5** and **6** inhibited the growth of a number of human cancer cell lines, including P388 leukemia, BXPC-3 pancreas, RPMI-7951 melanoma, U251 CNS, KAT-4 thyroid, NCI-H460 lung NSC, FADU pharynx and DU-145 prostate, with GI_50_ values ranging from 1.6 to 4.9 µg/mL. In addition, disc diffusion assays showed the ability of compound **6** to inhibit the growth of the Gram-positive bacterium *Micrococcus luteus*, with minimum inhibitory concentration (MIC) 25–50 µg/disk [19].

**Figure 4:**
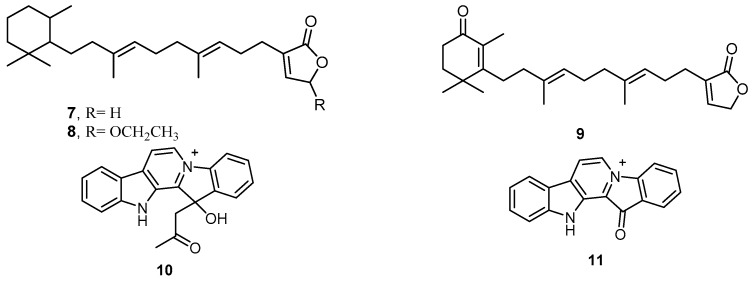
Chemical structures of compound **7**, compound **8**, isodehydroluffariellolide (**9**), homofascaplysin A (**10**) and fascaplysin (**11**).

Fractionation of the dichloromethane extract of the marine sponge *H. erectus*, collected from Fiji, led to the isolation of the two sesterterpenes **7** and **8**, isodehydroluffariellolide (**9**), homofascaplysin A (**10**), and fascaplysin (**11**) (Figure 4). Biological evaluation of these isolated compounds revealed activities for compounds **10** and **11**, both being potently active in vitro against the causative agent of tropical malaria *Plasmodium falciparum* strain K1 with IC_50_ values of 14 and 50 ng/mL, respectively, and against *P. falciparum* strain NF54 with IC_50_ values of 24 and 34 ng/mL, respectively [20]. Thus, homofascaplysin A (**10**), and fascaplysin (**11**) could serve as promising antimalarial agents for future work.

**Figure 5:**
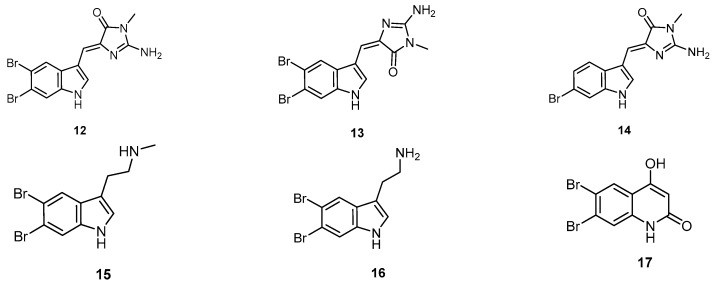
Chemical structures of the indole alkaloids (**12**–**16**) and compound **17**.

Five indole alkaloids **12**–**16** were isolated from the marine sponge *H. erectus*, which has been collected at Iriomote-Island, Okinawa Prefecture, Japan. Furthermore, the quinolone **17** was also isolated from the same marine sponge (Figure 5). The indole alkaloids **12**–**16** showed 100% selective inhibitory activity against the neuronal isozyme of nitric oxide synthase at a concentration of 125 µg/mL [12].

**Figure 6:**
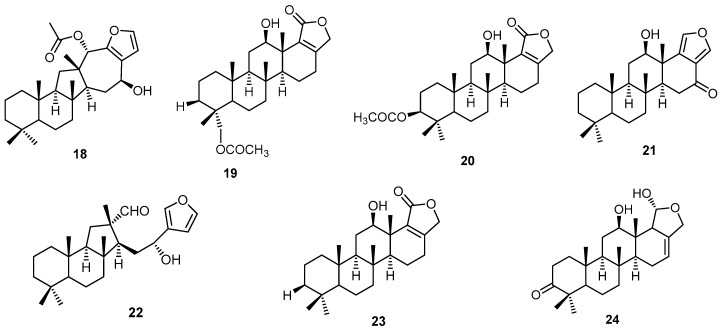
Chemical structures of salmahyrtisol A (**18**), 3-acetyl sesterstatin (**19**), 19-acetyl sesterstatin 3 (**20**), salmahyrtisol B (**21**), hyrtiosal (**22**), scalarolide (**23**) and salmahyrtisol C (**24**).

A pentacyclic sesterterpene ester salmahyrtisol A (**18**), three scalarane-type sesterterpenes, 3-acetyl sesterstatin (**19**), 19-acetyl sesterstatin 3 (**20**), and salmahyrtisol B (**21**), together with sesterterpenes hyrtiosal (**22**), scalarolide (**23**), and salmahyrtisol C (**24**) were obtained from the methanol extract of the Red Sea sponge *H. erectus* collected from a depth of 15–20 m from El Quseir, 120 km south of Hurghada, Egypt (Figure 6). Compounds **18**–**21** showed significant in vitro cytotoxicity against murine leukemia, human lung carcinoma, and human colon carcinoma, with IC_50_ values ≥1 µg/mL [16].

**Figure 7:**
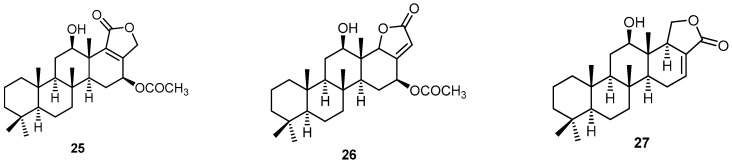
Chemical structures of sesterstatin 7 (**25**), 16-*epi*-scalarolbutenolide (**26**) and 25-dehydroxy-12-*epi*-deacetylscalarin (**27**).

The scalarane-type pentacyclic sesterterpene sesterstatin 7 (**25**) has been isolated from the marine sponge *H. erectus*, collected by hand using scuba at a depth of 15 m off Safaga at the Egyptian Red Sea coast, along with 16-*epi*-scalarolbutenolide (**26**), 25-dehydroxy-12-*epi*-deacetylscalarin (**27**) and 3-acetylsesterstatin 1 (**20**) (Figure 7). Compound **25** displayed 63% growth inhibition of *Mycobacterium tuberculosis* (H_37_Rv) (ATCC 27294) at a concentration of 6.25 µg/mL. Compound **26** showed moderate antimycobacterial activity (40% inhibition at 6.25 µg/mL), while compounds **27** and **20** exhibited weak activities at the same concentration [38].

**Figure 8:**
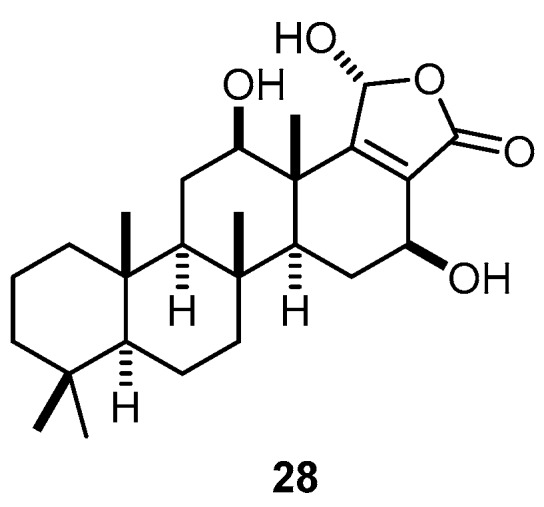
Chemical structure of sesterstatin 6 (**28**).

Sesterstatin 6 (**28**), another scalarane-type pentacyclic sesterterpene, was isolated from the Republic of Maldives marine sponge *H. erectus* (Figure 8). Compound **28** exhibited significant anticancer activity against murine P388 lymphocytic leukemia (ED_50_, effective dose causing 50% of the desired activity, 0.17 µg/mL) and a series of human tumor cell lines (GI_50_, concentration causing 50% of growth inhibition of cell proliferation, 0.18–89 µg/mL) [39].

**Figure 9:**
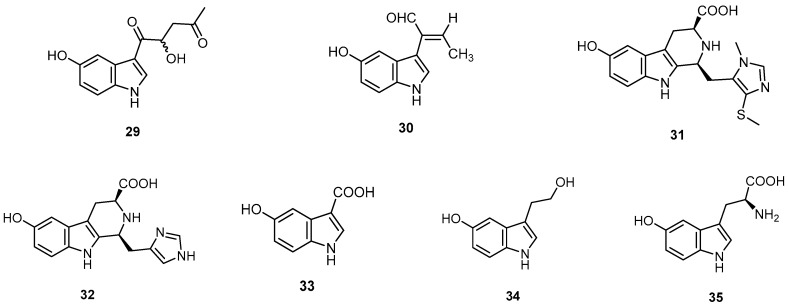
Chemical structures of hainanerectamine A (**29**), hainanerectamine B (**30**), hainanerectamine C (**31**) and the alkaloids **32**–**35**.

Hainanerectamines A (**29**) and B (**30**), two new indole alkaloids, and hainanerectamine C (**31**), a new β-carboline alkaloid, together with five known alkaloids (**1**, **32**–**35**), were isolated from the Hainan marine sponge *H. erectus* collected off Lingshui Bay, Hainan Province, China (Figure 9). Compounds **30**–**32** showed moderate inhibitory activity against Aurora A, a member of the serine/threonine kinase family involving in the regulation of cell division and a new target in cancer treatment, with IC_50_ values of 24.5, 13.6, and 18.6 μg/mL, respectively [14].

**Figure 10:**
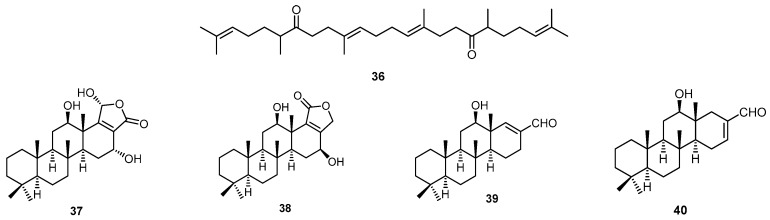
Chemical structures of the diketotriterpenoid **36**, hyrtiolide (**37**), 16-hydroxyscalarolide (**38**), 12-deacetyl-∆^17^-hyrtial (**39**) and 12-deacetylhyrtial (**40**).

An acyclic diketotriterpenoid (**36**) was isolated from the marine sponge *H. erectus* collected in Indonesia [40]. Three scalarane sesterterpenoids, hyrtiolide (**37**), 16-hydroxyscalarolide (**38**), and 12-deacetyl-∆^17^-hyrtial (**39**), along with scalarolide (**23**) and 12-deacetylhyrtial (**40**) were isolated from the marine sponge *H. erectus* collected from the coral reef of Ishigaki Island, Okinawa, Japan (Figure 10). Compounds **39** and **40** showed antiproliferative activity towards KB cells with IC_50_ values of 2.82 and 10 µg/mL, respectively [17].

**Figure 11:**
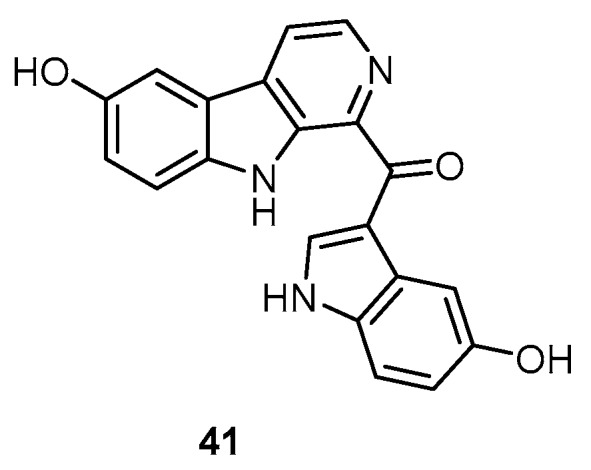
Chemical structure of hyrtiosulawesine (**41**).

Hyrtiosulawesine (**41**), a β-carboline alkaloid, together with 5-hydroxyindole-3-carbaldehyde (**1**), hyrtiosin B (**3**), and 5-hydroxy-3-(2-hydroxyethyl)indole (**34**) were isolated from the Indonesian specimens of a *H. erectus* collected from the northwest side of Lankai Island, off Makassar, South West Sulawesi (Figure 11) [15].

**Figure 12:**
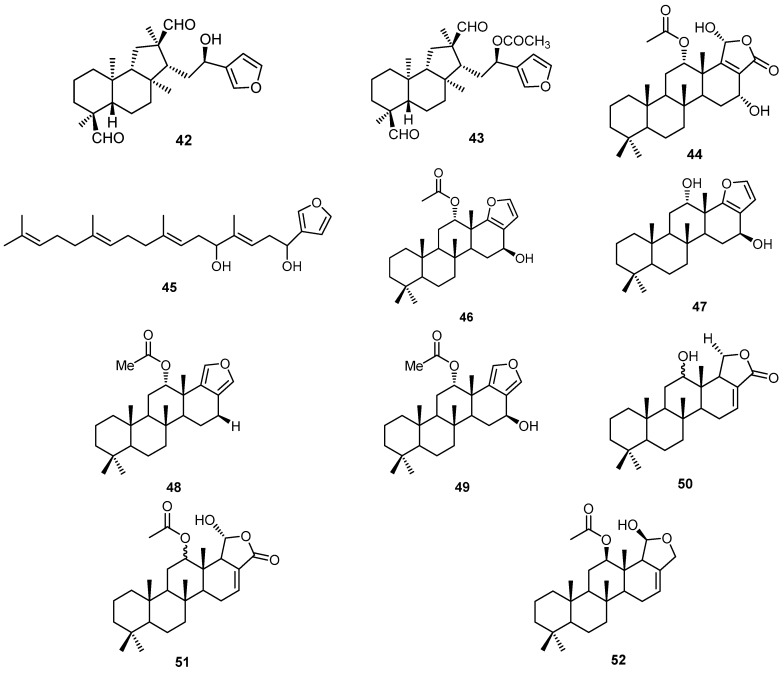
Chemical structures of 20-formylhyrtiosal (**42**), 16-*O*-acetyl-20-formylhyrtiosal (**43**), 12-α-*O*-acetylhyrtiolide (**44**), 5,10-dihydroxyfurospinulosine-1 (**45**) and compounds **46**–**52**.

Eleven sesterterpenes, 20-formylhyrtiosal (**42**), 16-*O*-acetyl-20-formylhyrtiosal (**43**), 12-α-*O*-acetylhyrtiolide (**44**), 5,10-dihydroxyfurospinulosine-1 (**45**), and compounds **46**–**52** have been isolated from the marine sponge *H. erectus* collected at Hainan, China (Figure 12) [41].

**Figure 13:**
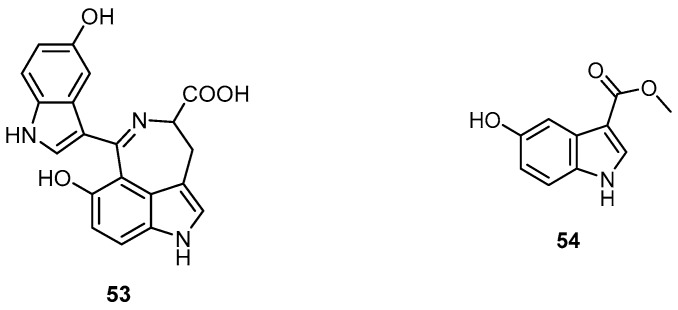
Chemical structures of hyrtiazepine (**53**) and compound **54**.

Fractionation of the methanolic extract of the marine sponge *H. erectus*, collected from Safaga at the Egyptian Red Sea coast, led to the isolation of the azepino-indole-type alkaloid hyrtiazepine (**53**) and 5-hydroxy-1*H*-indole-3-carboxylic acid methyl ester (**54**) (Figure 13), together with the known compounds hyrtiosulawesine (**41**), 5-hydroxyindole-3-carbaldehyde (**1**), hyrtiosin A (**2**), and hyrtiosin B (**3**). Hyrtiosulawesine (**41**) exhibited antiphospholipase A_2_ activity with an IC_50_ value of 14 µM in a fluorometric assay using *Crotalus adamanteus* venom phospholipase A_2_ [13].

**Figure 14:**
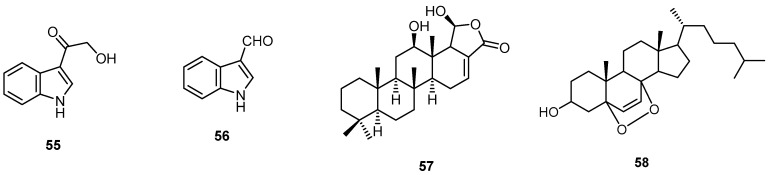
Chemical structures of deoxyhyrtiosine A (**55**), indole-3-carbaldehyde (**56**), 12-*O*-deacetyl-12-*epi*-scalarine (**57**) and 5α,8α-epidioxy-cholesta-6-en-3β-ol (**58**).

Deoxyhyrtiosine A (**55**) and indole-3-carbaldehyde (**56**) were isolated for the first time from the marine sponge *H. erectus* collected from the Red Sea in Egypt. In addition, the four known indoles; 5-hydroxy-1*H*-indole-3-carboxylic acid methyl ester (**54**), 5-hydroxy-1*H*-indole-3-carbaldehyde (**1**) and hyrtiosine A (**2**) were obtained. Three scalarane sesterterpenes, 16-hydroxyscalarolide (**38**), scalarolide (**23**) and 12-*O*-deacetyl-12-epi-scalarine (**57**), as well as 5α,8α-epidioxy-cholesta-6-en-3β-ol (**58**) were also isolated (Figure 14). Compounds **38, 54** and **56** exhibited growth inhibition activity against the L5178Y mouse lymphoma cell line, while compounds **1**, **38** and **55** showed mild antimicrobial activities against the Gram-positive bacterium *Bacillus subtilis* and the fungus *Saccharomyces cerevisiae* [21].

**Figure 15:**
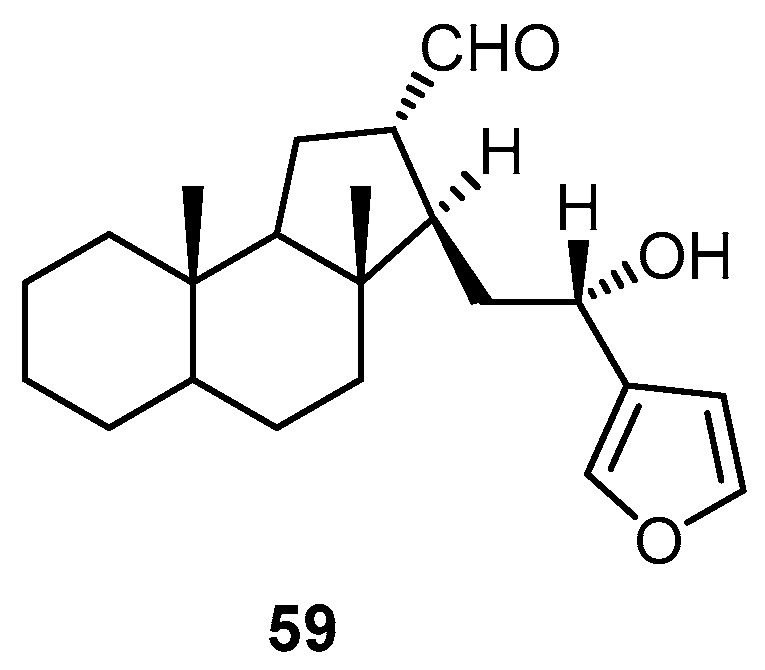
Chemical structure of hyrtiosal (**59**).

Hyrtiosal (**59**), isolated from the marine sponge *H. erectus* collected from Kerama Islands, Okinawa, inhibited HIV-1 integrase binding to viral DNA with an IC_50_ of 9.60 ± 0.86 µM (Figure 15) [22].

**Figure 16:**
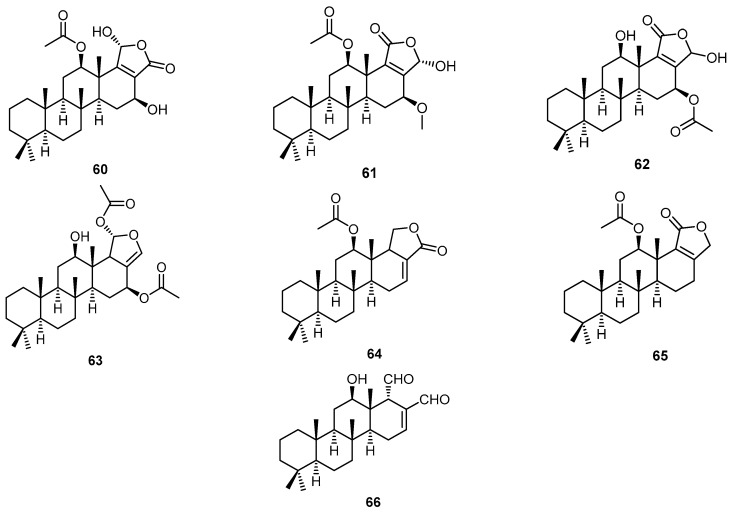
Chemical structures of 12-acetoxy,16-*epi*-hyrtiolide (**60**), 12β-acetoxy,16β-methoxy,20α-hydroxy-17-scalaren-19,20-olide (**61**) and the sesterterpenes **62**–**66**.

Two new sesterterpene analogs, 12-acetoxy,16-*epi*-hyrtiolide (**60**) and 12β-acetoxy,16β-methoxy,20α-hydroxy-17-scalaren-19,20-olide (**61**), together with seven previously reported scalarane-type sesterterpenes, **23**, **25**, and **62**–**66**, were isolated from the sponge *H. erectus* collected from the Red Sea, Egypt (Figure 16). The isolated compounds **25** and **60**–**66** showed considerable in vitro anticancer activity against breast adenocarcinoma (MCF-7), colorectal carcinoma (HCT-116) and hepatocellular carcinoma cells (HepG2), with IC_50_ values of 0.7–57.5 µM [42].

## 3. *H. reticulatus*

**Figure 17:**
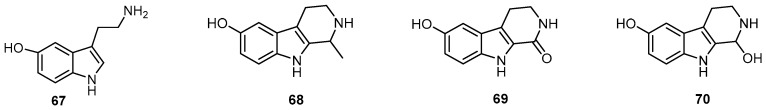
Chemical structures of serotonin (**67**) and compounds **68**–**70.**

Serotonin (**67**), 6-hydroxy-1-methyl-1,2,3,4-tetrahydro-β-carboline (**68**), 6-hydroxy-3,4dihydro-1-oxo-β-carboline (**69**) and 1,6-dihydroxy-1,2,3,4-tetrahydro-β-carboline (**70**) were isolated from the Indonesian specimens of the marine sponges *H. reticulatus*, collected from the west side of Bone Lola Reef, off Makassar, South West Sulawesi, Indonesia (Figure 17) [15].

**Figure 18:**
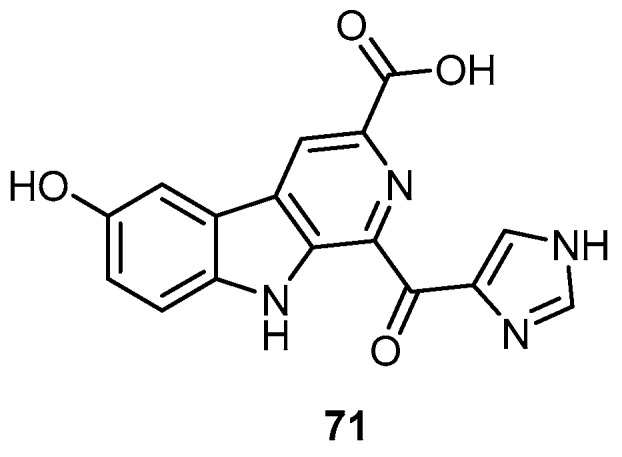
Chemical structure of hyrtiocarboline (**71**).

Hyrtiocarboline (**71**), 1-imidazoyl-3-carboxy-6-hydroxy-β-carboline alkaloid, were derived from a Papua New Guinea marine sponge, *H. reticulatus* (Figure 18). Compound **71** exhibited selective anticancer activity against H522-T1 non-small cell lung, MDA-MB-435 melanoma, and U937 lymphoma cancer cell lines, with IC_50_ values of 1.2, 3.0 and 1.5 µg/mL, respectively [23].

**Figure 19:**
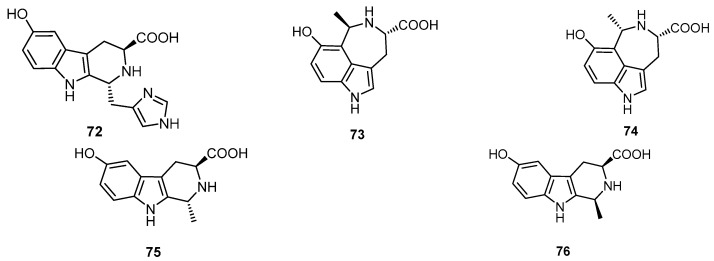
Chemical structures of hyrtioreticulin A (**72**), hyrtioreticulins C-E (**73**–**75**) and hyrtioerectine B (**76**).

Hyrtioreticulin A (**72**), hyrtioreticulin B (**32**) and hyrtioreticulins C-E (**73**–**75**) were identified in the marine sponge *H. reticulatus*, collected at a depth of 10 m in North Sulawesi, Indonesia, along with a known alkaloid, hyrtioerectine B (**76**) (Figure 19). The tetrahydro-β-carboline alkaloids hyrtioreticulins A (**72**) and B (**32**) inhibited ubiquitin-activating enzyme (E1) with IC_50_ values of 0.75 and 11 µg/mL, respectively. Interestingly, only five E1 inhibitors, panapophenanthrine, himeic acid A, largazole, and hyrtioreticulins A and B (**72** and **32**), were isolated from natural sources and, among them, compound **72** is the most potent E1 inhibitor [24].

**Figure 20:**
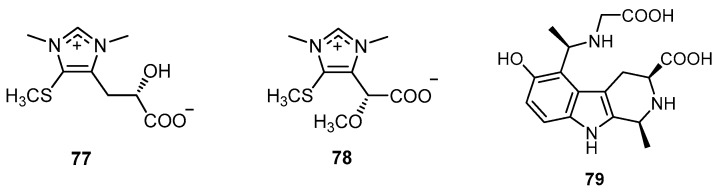
Chemical structures of reticulatin A (**77**), reticulatin B (**78**) and hyrtioreticulin F (**79**).

Two new 1,3-dimethyl-5-(methylthio)imidazolium alkaloids, reticulatins A (**77**) and B (**78**), and a new indole alkaloid hyrtioreticulin F (**79**) were isolated from the water-soluble fraction of the ethanol extract of the Indonesian marine sponge *H. reticulatus* (Figure 20) [25].

## 4. *H. gumminae*

**Figure 21:**
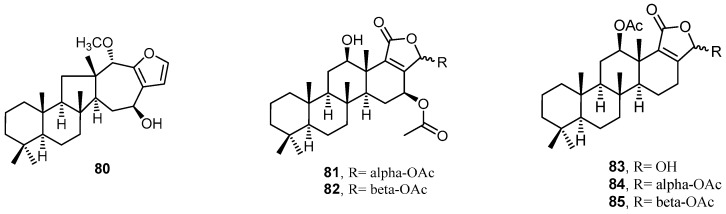

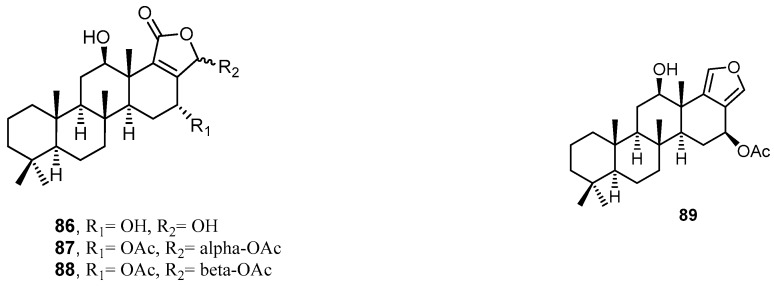
Chemical structures of similan A (**80**) and compounds **81**–**89**.

Chemical investigation on the ethylacetate-soluble fraction of the methanol extract of the marine sponge *H. gumminae* collected from Similan Island in the Andaman Sea, Thailand, yielded four sesterterpenoids, 12β,20-dihydroxy-16β-acetoxy-17-scalaren-19,20-olide (**62**), similan A (**80**), 12β-acetoxy20-hydroxy-17-scalaren-19,20-olide (**83**), and 12β,16α,20-trihydroxy-17-scalaren-19,20-olide (**86**), along with hyrtiosal (**22**), 12-epi-*O*-deacetyl-19-deoxyscalarin (**27**), hyrtiolide (**37**), and compounds **81**, **82**, **84**, **85**, and **87**–**89** (Figure 21). Some of these isolated compounds were tested for their in vitro anticancer activity against HuCCA-1 (human cholangiocarcinoma), KB (human epidermoid carcinoma of the mouth), HeLa (human cervical carcinoma), MDA-MB-231 (hormone-independent breast cancer), T47D (hormone-dependent breast cancer), and H69AR (multidrug-resistant small-cell lung cancer). Compounds **22** and **27** showed moderate anticancer activities (IC_50_ values of 5.2–57 µM) [26].

## 5. *H. communis*

**Figure 22:**
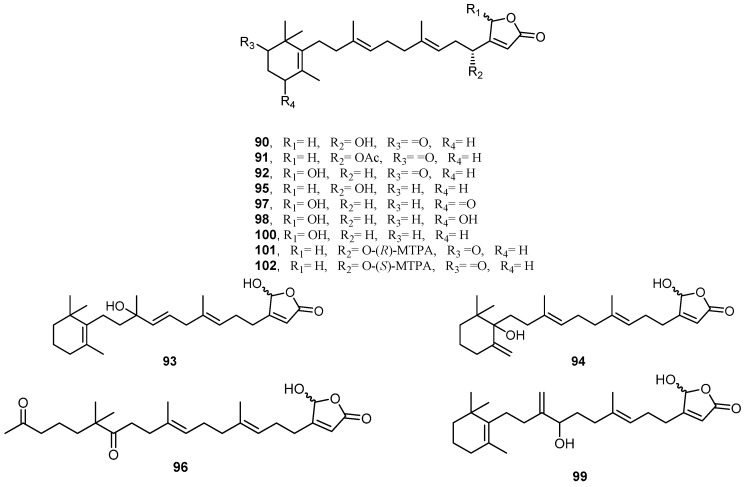
Chemical structures of the sesterterpenes **90**–**102**.

The extract of marine sponge *H. communis*, collected from a depth of 18–21 m from the northern reefs region off the coast of Palau, was found to inhibit transcription factor hypoxia-inducible factor-1 (HIF1) activation in T47D human breast tumor cells. Bioassay-guided fractionation of the *H. communis* extract led to the isolation and identification of the sesterterpenes **90**–**102** (Figure 22). Thorectidaeolide A (**90**), 4-acetoxythorectidaeolide A (**91**) and luffariellolide (**100**) showed potent inhibition activities of HIF-1 activation, with IC_50_ values of 3.2, 3.5, and 3.6 µM, respectively. Compound **100** exhibited a significant cytotoxic activity, which can be explained by its HIF-1 inhibitory activity [43].

## 6. *H. tubulatus*

**Figure 23:**
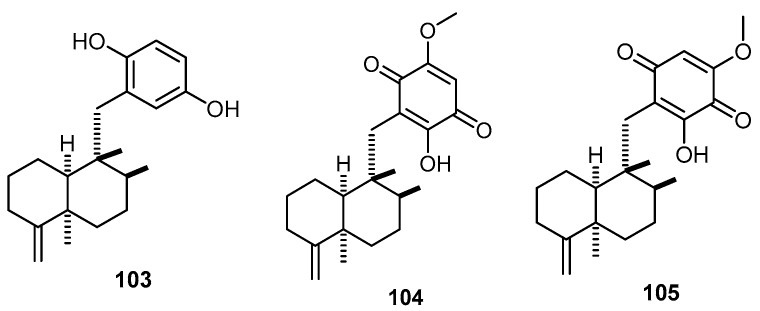
Chemical structures of arenarol (**103**), 5-epiilimaquinone (**104**) and 21-hydroxy-19-methoxyarenarone (**105**).

Arenarol (**103**) together with 5-epiilimaquinone (**104**) and 21-hydroxy-19-methoxyarenarone (**105**), which bear the 4,9-friedodrim-4(15)-ene skeleton, were isolated from the marine sponge *H. tubulatus* (which is currently identified as *Dysidea tubulata*) collected at a depth of 34.9 m by scuba diving off the South coast of Curaçao, Netherlands Antilles (Figure 23) [44].

## 7. Undescribed Marine Sponges of the Genus *Hyrtios*

**Figure 24:**
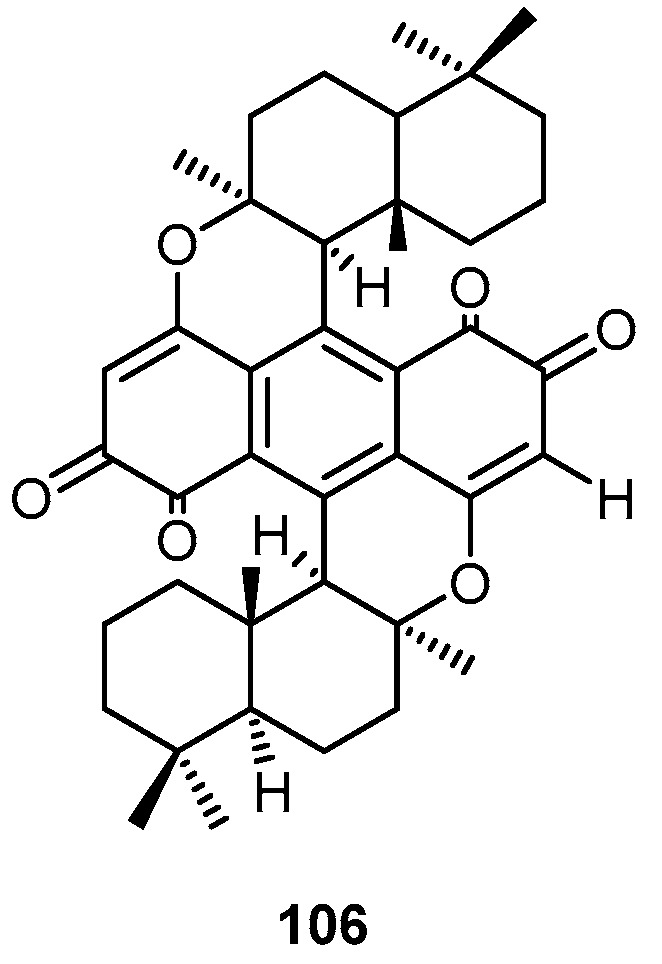
Chemical structure of dipuupehedione (**106**).

Dipuupehedione (**106**) has been isolated from the dichloromethane extract of a Caledonian marine sponge *Hyrtios* sp. (Figure 24), collected by SCUBA diving in New Caledonia (East Coast). Compound **106** showed significant cytotoxic activity on KB cells with IC_50_ value of 3 µg/mL [27].

**Figure 25:**
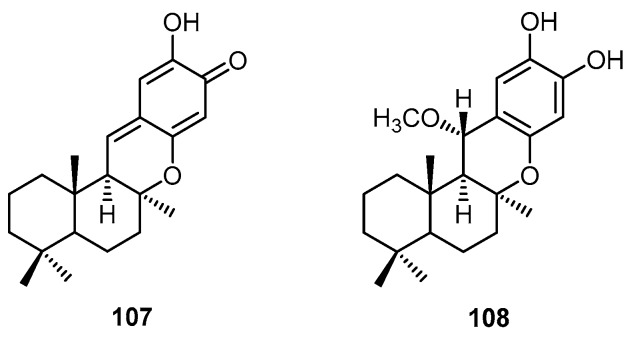
Chemical structures of puupehenone (**107**) and 15α-methoxypuupehenol (**108**).

Fractionation and chemical investigation of the dichloromethane extract of the marine sponge *Hyrtios* sp. collected from the East Coast of New Caledonia afforded dipuupehedione (**106**), puupehenone (**107**) and 15α-methoxypuupehenol (**108**) (Figure 25). Compound **108** exhibited similar antimicrobial and antifungal activity as puupehenone (**107**) and a lower cytotoxity towards KB cells with ED_50_ values of 6 and 0.5 µg/mL, respectively. Compound **108** exhibited slightly higher in vitro antimalarial activity than puupehenone **107**, against three strains of *Plasmodium falciparum* [28].

**Figure 26:**
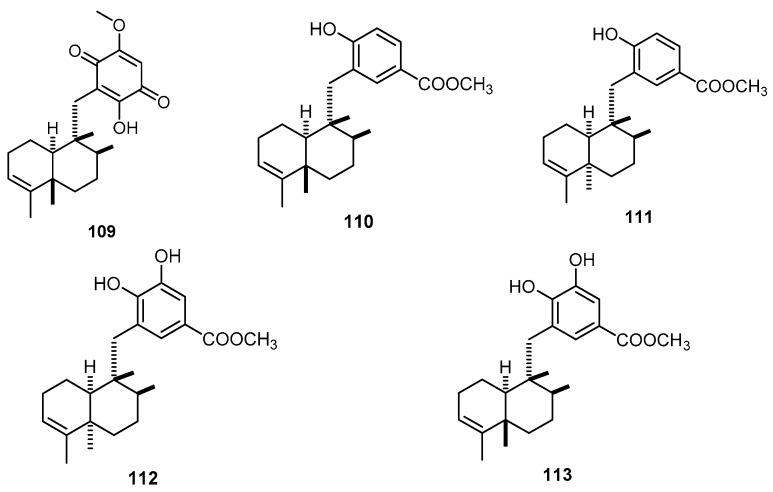
Chemical structures of isospongiaquinone (**109**), hyrtiophenol (**110**), 5-epihyrtiophenol (**111**), 18-hydroxy-5-epihyrtiophenol (**112**) and 18-hydroxyhyrtiophenol (**113**).

The methanolic extract of a marine sponge of the genus *Hyrtios*, collected at a depth of 35–45 m by dredging off Mahé (Seychelles Islands), was found to contain isospongiaquinone (**109**) together with four compounds with a 4,9-friedodrim-3-ene skeleton, hyrtiophenol (**110**), 5-epihyrtiophenol (**111**), 18-hydroxy-5-epihyrtiophenol (**112**) and 18-hydroxyhyrtiophenol (**113**) (Figure 26) [44].

**Figure 27:**
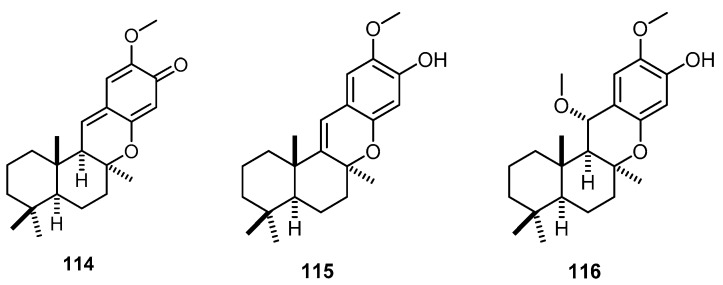
Chemical structures of compounds **114**–**116.**

Chemical Investigations on an Indonesian *Hyrtios* sp. collected from Togian Island in Tomini Bay, north Sulawesi, Indonesia, led to the isolation and identification of three compounds, (+)-(5*S*,8*S*,9*R*,10*S*)-20-methoxypuupehenone (**114**), (+)-(5*S*,8*S*,10*S*)-20-methoxy-9,15-ene-puupehenol (**115**) and (+)-(5*S*,8*S*,9*R*,10*S*)-15,20-dimethoxypuupehenol (**116**) (Figure 27) [29].

**Figure 28:**
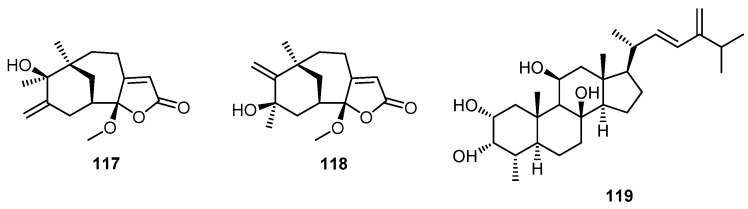
Chemical structures of hyrtiosenolide A (**117**), hyrtiosenolide B (**118**) and hyrtiosterol (**119**).

Two sesquiterpene γ-methoxybutenolides, hyrtiosenolide A (**117**) and hyrtiosenolide B (**118**), along with a 4α-methyl polyoxygenated steroid, hyrtiosterol (**119**), were obtained from a marine sponge of the genus *Hyrtios* collected from the Red Sea, Hurghada, Egypt (Figure 28). Hyrtiosenolides A (**117**) and B (**118**) displayed weak in vitro antibacterial activity against *Escherichia coli*. An inhibition zone of 7 mm was observed when 100 µg of **117** or **118** was applied to a 6 mm diameter paper disk on an agar plate inoculated with *E. coli* [45].

**Figure 29:**
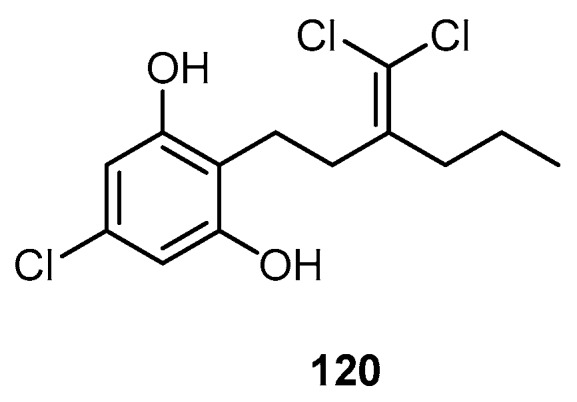
Chemical structure of poipuol (**120**).

A trichlorinated metabolite, poipuol (**120**), was isolated from an undescribed marine sponge *Hyrtios* sp. collected in Kauai Island, Hawaii (Figure 29) [46].

**Figure 30:**
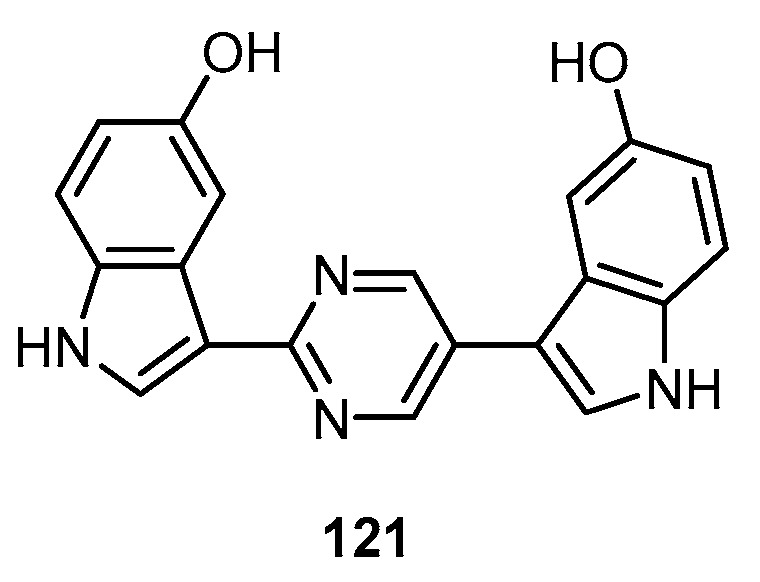
Chemical structure of hyrtinadine A (**121**).

Hyrtinadine A (**121**), a cytotoxic bis-indole alkaloid with a pyrimidine moiety, was isolated from a marine sponge *Hyrtios* sp. collected off Unten-Port, Okinawa (Figure 30). Compound **121** was the first example of a bis-indole alkaloid with a 2,5-disubstituted pyrimidine ring between two indole rings. Hyrtinadine A (**121**) showed in vitro cytotoxic activity against murine leukemia L1210 cells with IC_50_ value of 1 µg/mL and against human epidermoid carcinoma KB cells with IC_50_ value of 3 µg/mL [47].

**Figure 31:**
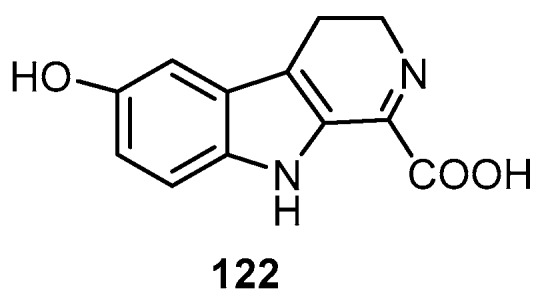
Chemical structure of compound **122**.

Biological and chemical investigations on the crude extract of the Micronesian marine sponge *Hyrtios* sp. resulted in the isolation of a new alkaloid, 1-carboxy-6-hydroxy-3,4-dihydro-β-carboline (**122**) (Figure 31), together with the known metabolites, 5-hydroxyindole-3-carbaldehyde (**1**), hyrtiosin A (**2**), hyrtiosin B (**3**), 5-hydroxy-1*H*-indole-3-carboxylic acid methyl ester (**54**), serotonin (**67**) and 6-hydroxy-3,4-dihydro-1-oxo-beta-carboline (**69**). Among these isolated compounds, hyrtiosin B (**3**) showed a potent inhibitory activity against isocitrate lyase (ICL) of *Candida albicans* with an IC_50_ value of 89.0 µM [48].

**Figure 32:**
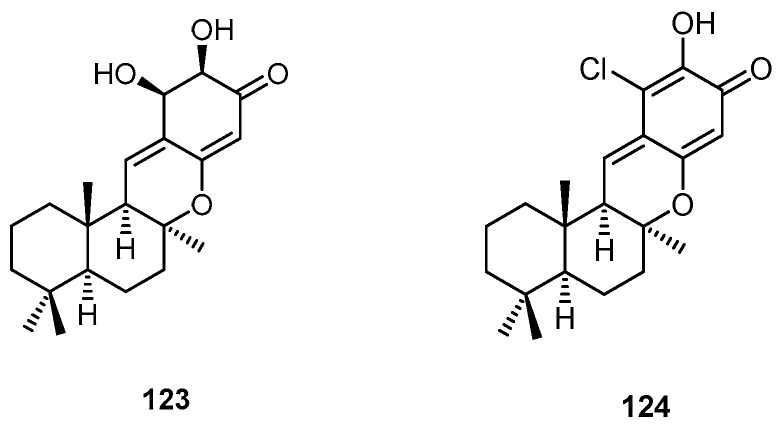
Chemical structures of puupehanol (**123**) and chloropuupehenone (**124**).

The sesquiterpene-dihydroquinone derivative puupehanol (**123**) and chloropuupehenone (**124**) were isolated from a marine sponge of the genus *Hyrtios* collected in Papua New Guinea, together with the known compound puupehenone (**107**) (Figure 32). Compound **107** showed potent antifungal activity against *Cryptococcus neoformans* and *Candida krusei* with minimum fungicidal concentration (MFC) values of 1.25 and 2.50 µg/mL, respectively [49].

**Figure 33:**
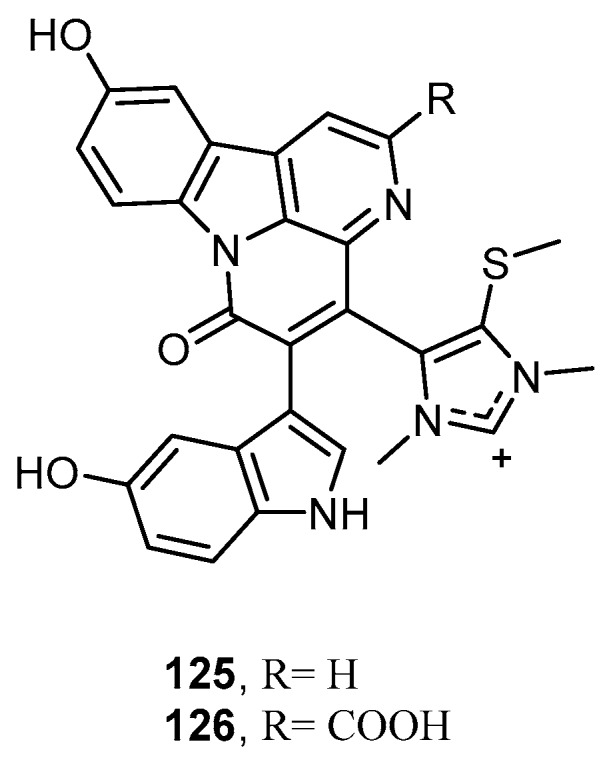
Chemical structures of hyrtimomine D (**125**) and hyrtimomine E (**126**).

Two structurally unique bisindole alkaloids possessing the canthin-6-one skeleton with a hydroxyindole and an imidazolium unit, namely hyrtimomine D (**125**) and hyrtimomine E (**126**), have been isolated from an Okinawan marine sponge *Hyrtios* sp. collected off Kerama Islands (Figure 33). Compounds **125** and **126** exhibited antifungal activity against *C. albicans* (IC_50_, 4 and 8 μg/mL, respectively) and *C. neoformans* (IC_50_, 4 and 8 μg/mL, respectively). Furthermore, hyrtimomine D (**125**) displayed inhibitory activity against the Gram-positive bacterium *Staphylococcus aureus* with MIC value of 4 μg/mL and against *Trichophyton mentagrophytes* with MIC value of 16 μg/mL [30].

**Figure 34:**
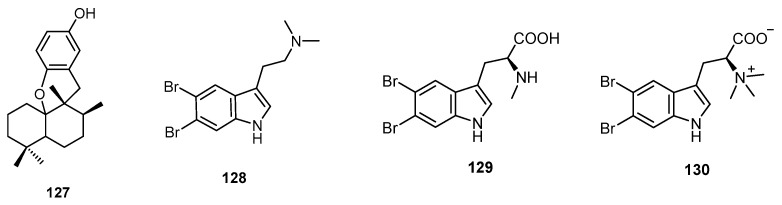
Chemical structures of aureol (**127**), *N*,*N*-dimethyl-5,6-dibromotryptamine (**128**), 5,6-dibromoabrine (**129**) and 5,6-dibromo-L-hypaphorine (**130**).

Chromatographic fractionation of the extracts of *Hyrtios* sp., collected from Fiji Islands, afforded aureol (**127**) together with five dibromoalkaloids (**15**, **16**, **128**–**130**). The structures of compounds **15**, **16**, and **128**–**130** were identified as *N*-methyl-5,6-dibromotryptamine (**15**), 5,6-dibromotryptamine (**16**), *N*,*N*-dimethyl-5,6-dibromotryptamine (**128**), 5,6-dibromoabrine (**129**) and 5,6-dibromo-l-hypaphorine (**130**) (Figure 34). The sesquiterpene aureol (**127**) exhibited potent antioxidant activity with an oxygen radical absorbance capacity (ORAC) value of 0.29, while compound **130** displayed a weak bee venom PLA2 inhibition (IC_50_ 0.2 mM) and an antioxidant activity with an ORAC value of 0.22 [31].

**Figure 35:**
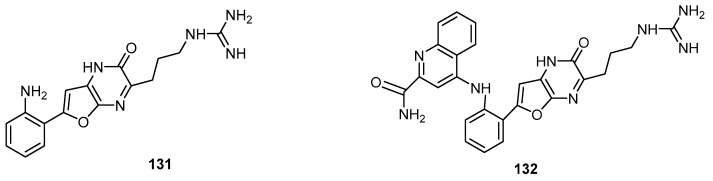
Chemical structures of hyrtioseragamine A (**131**) and hyrtioseragamine B (**132**).

The two new alkaloids hyrtioseragamine A (**131**) and hyrtioseragamine B (**132**), the first natural products possessing a furo[2,3-*b*]pyrazin-2(1*H*)-one moiety and a guanidino group, were isolated from an marine sponge *Hyrtios* sp. collected off Seragaki, Okinawa (Figure 35). Compounds **131** and **132** exhibited antifungal activities against *Aspergillus niger* with MIC values of 8.33 and 16.6 μg/mL, respectively, and against *Cryptococcus neoformans* with MIC values of 33.3 and 16.6 μg/mL, respectively. However, compounds **131** and **132** did not show in vitro cytotoxic activity against murine lymphoma L1210 and human epidermoid carcinoma KB cells (IC_50_ > 10 μg/mL) [32].

**Figure 36:**
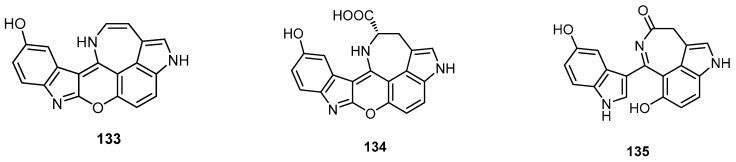
Chemical structures of hyrtimomine A (**133**), hyrtimomine B (**134**) and hyrtimomine C (**135**).

Hyrtimomine A (**133**) and hyrtimomine B (**134**), new heteroaromatic alkaloids possessing a fused hexacyclic 6/5/6/6/7/5 ring system, and hyrtimomine C (**135**), a new alkaloid consisting of hydroxyindole and azepino-hydroxyindole moieties, were discovered from an Okinawan marine sponge *Hyrtios* sp. collected off Kerama Islands, Okinawa (Figure 36). Hyrtimomine A (**133**) showed in vitro cytotoxic activity against human epidermoid carcinoma KB cells (IC_50_ = 3.1 μg/mL) and murine leukemia L1210 cells (IC_50_ = 4.2 μg/mL), while compounds **134** and **135** did not show cytotoxic activity (IC_50_ > 10 μg/mL) [33].

**Figure 37:**
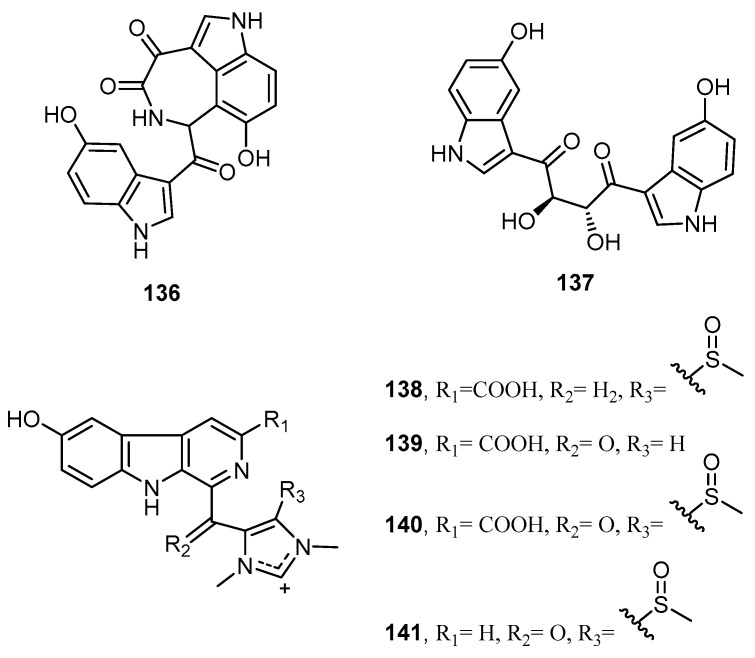
Chemical structures of hyrtimomines F–K (**136**–**141**).

A new structurally unique bisindole alkaloid possessing an α-keto-ɛ-caprolactam ring, hyrtimomine F (**136**), a new symmetrical bisindole alkaloid, hyrtimomine G (**137**), and four new indole alkaloids possessing β-carboline skeleton with an imidazolium unit, hyrtimomines H–K (**139**–**141**), were isolated from Okinawan marine sponges *Hyrtios* spp. collected at Kerama Islands (Figure 37). Compounds **136**, **137**, and **139** showed inhibitory effects against *A. niger* with IC_50_ value of 8 µg/mL, while **139** displayed inhibitory effect against *C. neoformans* with IC_50_ of 4 µg/mL [34].

**Figure 38:**
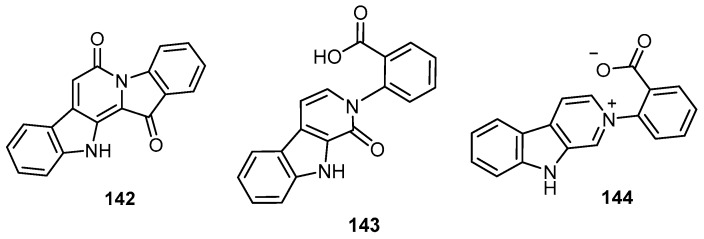
Chemical structures of 6-oxofascaplysin (**142**), secofascaplysic acid (**143**) and reticulatate (**144**).

Mass-guided fractionation of the methanolic extract from a specimen of the Australian marine sponge *Hyrtios* sp. led to the isolation of two tryptophan alkaloids, 6-oxofascaplysin (**142**), and secofascaplysic acid (**143**), in addition to the two metabolites fascaplysin (**11**) and reticulatate (**144**) (Figure 38). Compounds **11** and **142**–**144** displayed in vitro cytotoxic activity against a prostate cancer cell line (LNCaP) with IC_50_ values ranging from 0.54 to 44.9 μM [35]. 

**Figure 39:**
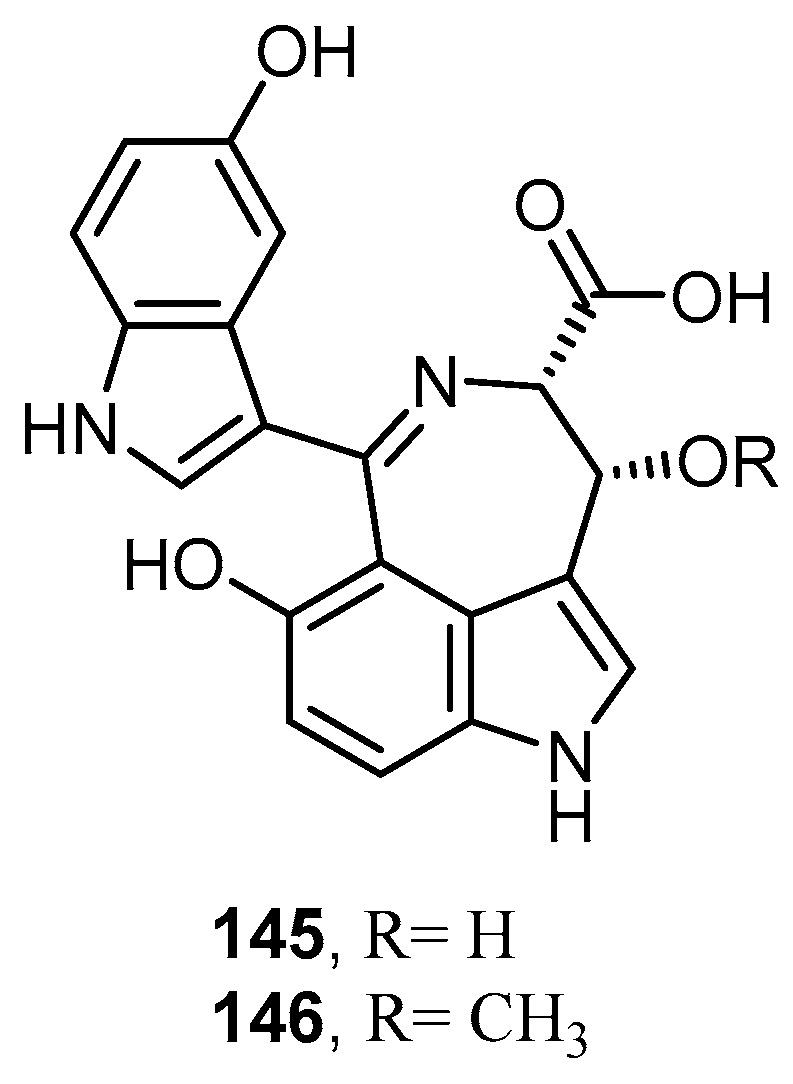
Chemical structures of hyrtinadine C (**145**) and hyrtinadine D (**146**).

Two new relatively rare bisindole alkaloids possessing a 3,4-fused azepinoindole skeleton, hyrtinadine C (**145**) and hyrtinadine D (**146**), were isolated from an Okinawan marine sponge *Hyrtios* sp. collected off Unten Port (Figure 39). Compound **145** showed antifungal activity against *A. niger* with IC_50_ value of 32 µg/mL, while compound **146** showed antibacterial activity against the Gram-negative bacterium *E. coli* with MIC value of 16 µg/mL and against the Gram-positive bacterium *B. subtilis* with MIC value of 16 µg/mL [36].

Genus *Hyrtios* attracted scientists’ attention and sparked high synthetic efforts for the synthesis of the isolated compounds from its members [37,38]. Several compounds have been synthesized such as Salmahyrtisol A from *Hyrtios erecta* [39,40], Similan A from the Thai sponge *Hyrtios gumininae* [41,42], sesterstatin 1 from *Hyrtios erecta* [43,44], Spongistatins 1 (Altohyrtin A ) from *Hyrtios erecta* [45,46], (−)-Hyrtiosal and its C-16 epimer have been synthetized from sclareol [47] which was previously isolated from the sponge *Hyrtios erectus* [48].

## 8. Conclusions

Marine sponges harbor a huge repertoire of yet undiscovered natural products possessing a broad-spectrum of pharmacological applications. Among the several *Hyrtios* species discovered, *H. erectus*, *H. reticulatus*, *H. gumminae*, *H. communis*, and *H. tubulatus* were the most prolific producers of secondary metabolites with various pharmaceutically and medically relevant bioactivities (Table 1). A total of 146 natural products from various marine sponges belonging to the genus *Hyrtios* were reported in MarinLit database until 2016 as well as in the literature*. H. erectus* represents the most frequently investigated source of bioactive natural products from *Hyrtios* sp. in terms of number of natural products isolated. The discovery of new species from the genus *Hyrtios* indicates that there is room for new natural products discovery.

With the currently available data a correlation between geographical area where the sponges were collected and the type of metabolites found for this particular species can be concluded. Sponges collected off Okinawa (Japan) were richer in alkaloids, especially indole alkaloids (indole alkaloids possessing β-carboline skeleton with an imidazolium unit, azepino-hydroxyindole moieties) and bisindole alkaloids. In addition, sponges collected off Fiji were rich with brominated alkaloids and sesterterpenes. Sponges collected from the Republic of Maldives were very rich in scalarane-type pentacyclic sesterterpene and sesterstatins. Furthermore, sponges collected off Indonesia are rich in β-carboline alkaloids. On the other hand, sponges collected off the Red sea were rich in terpenoids, especially sesterterpenes, sesterstatins as well as indole alkaloids and azepino alkaloids, with the majority of the isolated compounds being terpenoids. The different geographical chemotypes might be explained by variations in the microbial community associated with the respective sponges. Sponges have developed intimate association with a huge diversity of microorganisms, such as viruses, bacteria, archaea, fungi, protozoa and single-celled algae. It is often unclear whether the compounds of interest are biosynthesized by the sponges or their associated microbes [50,51]. Many bioactive natural products from marine invertebrates have striking similarities to metabolites of their associated microorganisms, especially bacteria [52,53,54,55,56,57]. In most cases, the development of sponge-derived drugs is challenged by environmental concerns and technical problems associated with harvesting large amounts of sponges. Sponge-associated microorganisms may represent a sustainable source of sponge-derived natural products that could be established through a symbiont culture or by transferring its biosynthetic genes into culturable microorganisms [58]. Based on available scientific literature, it is evident that marine sponges within genus *Hyrtios* represent a rich source of natural products with various biological activities.

## Figures and Tables

**Table 1:** Secondary metabolites isolated from marine sponges from the genus *Hyrtios*.

Name of *Hyrtios* sp.	Number of Secondary Metabolites	Bioactivity	Reference
*Hyrtios erectus*	66	In vitro cytotoxic activity against human epidermoid carcinoma KB cells. Immunosuppressive activity in the B-lymphocytes reaction assay. Inhibited the growth of a number of human cancer cell lines, including P388 leukemia, BXPC-3 pancreas, RPMI-7951 melanoma, U251 CNS, KAT-4 thyroid, NCI-H460 lung NSC, FADU pharynx and DU-145 prostate. Inhibit the growth of the Gram-positive bacterium *Micrococcus luteus*. Antimalarial agents. Showed 100% selective inhibitory activity against the neuronal isozyme of nitric oxide synthase. Showed significant in vitro cytotoxicity against murine leukemia, human lung carcinoma, and human colon carcinoma. Antimycobacterial activity. Exhibited significant anticancer activity against murine P388 lymphocytic leukemia and a series of human tumor cell lines. Inhibitory activity against Aurora A Antiproliferative activity towards KB cells Antiphospholipase A_2_ activity. Growth inhibition activity against the L5178Y mouse lymphoma cell line, Antimicrobial activities against the Gram-positive bacterium *Bacillus subtilis* and the fungus *Saccharomyces cerevisiae*. In vitro anticancer activity against breast adenocarcinoma (MCF-7), colorectal carcinoma (HCT-116) and hepatocellular carcinoma cells (HepG2).	[1] [2] [3] [4] [5] [6] [7] [8] [9] [10] [11] [12] [13] [14] [15] [16] [17]
*Hyrtios reticulatus*	13	Selective anticancer activity against H522-T1 non-small cell lung, MDA-MB-435 melanoma, and U937 lymphoma cancer cell lines. Inhibited ubiquitin-activating enzyme (E1).	[12] [18] [19] [20]
*Hyrtios gummina*	10	In vitro anticancer activity against HuCCA-1 (human cholangiocarcinoma), KB (human epidermoid carcinoma of the mouth), HeLa (human cervical carcinoma), MDA-MB-231 (hormone-independent breast cancer), T47D (hormone-dependent breast cancer), and H69AR (multidrug-resistant small-cell lung cancer).	[21]
*Hyrtios communis*	13	Inhibit transcription factor hypoxia-inducible factor-1 (HIF1) activation in T47D human breast tumor cells. Significant cytotoxic activity.	[22]
*Hyrtios tubulatus* currently identified as *Dysidea tubulata*)	3	No biological activities have been reported	[23]
Undescribed marine sponges of the genus *Hyrtios*	41	Antimicrobial and antifungal activity. In vitro antimalarial activity. Antibacterial activity against *Escherichia coli*. In vitro cytotoxic activity against murine leukemia L1210 cells and against human epidermoid carcinoma KB cells. A potent inhibitory activity against isocitrate lyase (ICL) of *Candida albicans*. Potent antifungal activity against *Cryptococcus neoformans* and *Candida krusei* with minimum fungicidal concentration (MFC). Antifungal activity against *C. albicans* and *C. neoformans* and against *Trichophyton mentagrophytes*. Potent antioxidant activity. Antifungal activities against *Aspergillus niger* and against *Cryptococcus neoformans*. In vitro cytotoxic activity against human epidermoid carcinoma KB cells and murine leukemia L1210 cells. Inhibitory effects against *A. niger* and inhibitory effect against *C. neoformans*. In vitro cytotoxic activity against a prostate cancer cell line. Antifungal activity against *A. niger*.	[24] [25] [23] [26] [27] [28] [29] [30] [31] [32] [33] [34] [35] [36] [37]
